# Effect of Wheat Varieties and Cultivation Environments on Grain Endophytes, Microbial Communities, and Quality of Medium-High Temperature Daqu in Chinese Baijiu

**DOI:** 10.3390/foods14060982

**Published:** 2025-03-13

**Authors:** Huixian Zhou, Mengmeng Zhao, Qinqin Xiong, Chengcheng Feng, Zhien Pu, Guoyue Chen, Songtao Wang, Yi Dong, Xiaojun Wang, Hai Long, Qiantao Jiang, Jirui Wang, Yuming Wei, Youliang Zheng, Wei Li

**Affiliations:** 1 College of Agronomy, Sichuan Agricultural University, Chengdu 611130, China; zhxzka0221@163.com (H.Z.); 13606750252@163.com (M.Z.); xiongqinqin816@163.com (Q.X.);; 2 Triticeae Research Institute, Sichuan Agricultural University, Chengdu 611130, China; 3 National Engineering Research Center of Solid-State Brewing, Luzhou 646000, China; 4 Chengdu Institute of Biology, Chinese Academy of Sciences, Chengdu 610041, China

**Keywords:** Chinese Baijiu starter, high-throughput sequencing, wheat grain endophytes, Daqu microbial community, correlation analysis

## Abstract

Wheat grain serves as the primary raw material for producing medium-high temperature (MT)-Daqu, a fermentation starter crucial for Chinese Baijiu production, characterized by spontaneous fermentation without the inoculation of exogenous substances. However, the interactions among wheat varieties, cultivation environments, and the resulting Daqu quality remain poorly understood. This study evaluates three wheat varieties harvested from three distinct cultivation environments, examining wheat grain quality, grain-associated endophytes, and physicochemical properties and microbial communities of MT-Daqu at 0, 9, and 90 days of fermentation. The results revealed the cultivation environment had the most pronounced impact on wheat fungal endophytes. The physicochemical properties of Daqu were primarily impacted by variety, namely, the enzyme activity impacted by environmental factors. *Pantoea*, *Aspergillus*, and *Stephylium* are key microbial genera shared between wheat grains and MT-Daqu. Redundancy analysis highlighted the critical roles of moisture content, starch content, and amino acid nitrogen levels in driving microbial succession in Daqu. Mantel analysis demonstrated significant correlations between the abundance of dominant fungal endophytes in wheat grains and Daqu quality parameters, including starch content (r = 0.45; *p* < 0.01), saccharifying activity (r = 0.41), liquefying activity (r = 0.31), and esterifying activity (r = 0.30) (*p* < 0.05). Spearman correlation analysis indicated that *Nesterenkonia*, *Aspergillus*, *Cryptococcus*, *Dioszegia*, *Golubevia*, *Udeniomyces* and *Stemphylium* are the dominant wheat-derived bacterial genera associated with the abundance of microorganisms in MT-Daqu. This study elucidated the “cultivation environment–grain endophyte–Daqu microorganism” microbial transmission pathway, providing a theoretical foundation for breeding wheat varieties optimized for Daqu production and identifying suitable production regions.

## 1. Introduction

Chinese Baijiu, one of the oldest distilled liquors in the world, holds a prominent place in traditional Chinese culture [1]. Based on processing technologies, Baijiu is categorized into four primary types: sauce flavor, strong flavor, light flavor, and mixed flavor [2]. Among these, strong-flavor Baijiu is particularly esteemed for its distinctive taste and rich aromatic profile [3]. A critical component in the production of strong-flavor Baijiu is medium-high temperature (MT)-Daqu, a saccharification and fermentation starter integral to its brewing process [4]. MT-Daqu is produced using wheat as the primary raw material, with barley, peas, and other grains serving as supplementary substrates. Its production relies on spontaneous fermentation, that is, the proliferation of naturally occurring microorganisms—such as molds, yeasts, and bacteria—sourced from raw materials, water, air, and the production environment, rather than through the addition of pure cultured strains [5]. Therefore, the raw materials, production techniques, and fermentation environment synergistically shape the microbial community’s composition and functionality, ultimately influencing the liquor’s flavor profile [6]. Notably, a recent study [7] has revealed that 94.7% of fungal species and 20.4% of bacterial species in Feng-flavor newly pressed Daqu originate from wheat, highlighting wheat’s critical role in shaping the microbial diversity of this fermentation starter.

Wheat (*Triticum aestivum* L.) serves as a crucial raw material for the fermentation of Daqu and Baijiu. Rich in carbohydrates, proteins and other nutrients, wheat provides essential carbon and nitrogen sources to support the growth and reproduction of microorganisms during fermentation [8]. The quality of wheat, characterized by traits such as starch content, protein levels, and grain hardness, is significantly affected by the interplay between wheat variety and cultivation environment [9]. These quality traits, in turn, have a profound impact on the performance and characteristics of wheat-based products [10]. For example, the ratio of amylose to amylopectin, which varies considerably among wheat varieties, plays a pivotal role in determining the quality of wheat-based foods such as bread and noodles [11]. Despite the extensive research on wheat quality and its impact on food products, there remains a significant knowledge gap regarding how wheat varieties and cultivation environments affect fermented products such as Daqu, liquor, and vinegar.

A key distinction between wheat-based fermented products and other traditional processed products, such as noodles and biscuits, lies in the critical role of endogenous microbial flora carried by the grains. Wheat grains host a substantial population of endophytes, such as *Pseudomonas* and *Bacillus*, which are vertically transmitted and conserved across successive plant generations [12,13]. The diversity and stability of seed endophytic microbial communities are shaped by wheat through selective screening and regulation, influenced by seed coat properties, plant-specific metabolites, and vascular transport mechanisms [14]. Therefore, although the parent plant contributes to shaping the endophytic community in its seeds, these microorganisms may also be acquired from the surrounding soil [15]. Given this intricate interaction, the influence of wheat variety and cultivation environment on grain endophytes—and consequently on Daqu quality—should not be overlooked. Current research on wheat seed endophytes primarily focuses on their role in enhancing wheat growth and development [16], improving stress resistance [17], and affecting grain quality. *Pseudomonas* and *Penicillium* have been confirmed to exhibit a significant positive correlation with the starch-to-protein ratio in wheat [18]. However, the influence of endophytic communities on processed products, particularly fermented products such as Daqu, remains largely unexplored. To bridge this knowledge gap, it is essential to investigate the relationship between wheat and Daqu quality, emphasizing the interplay between grain endophytes and microbial communities in Daqu. A deeper understanding of these relationships will not only aid in improving wheat varieties for fermentation purposes but also contribute to the production of high-quality Daqu and Baijiu.

In this study, MT-Daqu was produced using raw grains from three wheat cultivars—B4361, Chuanmai 39, and Lunuomai 3—grown in Chongzhou, Mianzhu, and Luzhou, China. The quality attributes and endophytes of the wheat grains, along with the quality and microbial communities of the Daqu, were comprehensively analyzed. Bioinformatics and multivariate statistical approaches were employed to investigate the effects of wheat varieties and cultivation environments on the quality and microbial traits of both wheat and Daqu. Correlation analyses were further conducted to elucidate the relationships among these traits. 

This study provides valuable insights into the mechanism underlying the “cultivation environment–grain endophyte–Daqu microorganism” continuum. The findings offer a theoretical foundation for the development of wheat cultivars specifically suited for Daqu production and the screening of suitable cultivation regions.

## 2. Materials and Methods

### 2.1. Plant Materials

Three wheat cultivars—B4361, Chuanmai 39 (CM39), and Lunuomai 3 (LNM3)—were selected as raw materials for Daqu production in this study. All seeds were provided by the Triticeae Research Institute, Sichuan Agricultural University, China. The cultivars were cultivated across three locations in Sichuan Province (China): Chongzhou (CZ; 30°37′ N, 103°40′ E), Mianzhu (MZ; 31°20′ N, 104°13′E), and Luzhou (LZ; 28°53′ N, 105°26′ E). Each cultivar was planted on approximately 5 acres per site in November 2019. Field management followed standard local agricultural methods. The wheat samples were harvested in May 2020 and sun-dried for three days in a dedicated drying field, using sunlight and natural wind. After drying, they were all stored in the same warehouse to ensure consistency prior to analysis.

### 2.2. Wheat Grain Collection

Wheat ears were harvested approximately 35 d after flowering, when the grains had reached maturity. The harvest was conducted in three separate batches on the same day: First batch: Using sterile gloves, the ears were carefully collected, threshed, and sealed in sterile plastic bags. These samples were stored at −80 °C for the analysis of endophytic microorganisms in the wheat grains. Second batch: Collected and sealed in sterile plastic bags following the same procedure, this batch was used to assess wheat grain quality parameters and seed vigor. Third batch: Harvested and stored in mesh bags, this batch was exclusively designated for Daqu preparation.

### 2.3. Daqu Preparation

The spontaneous solid-state fermentation of MT-Daqu starters was conducted at the Luzhou Laojiao Daqu Production Ecological Garden, operated by Luzhou Laojiao Co. Ltd., in Luzhou, Sichuan, China (28°90′ N, 105°43′ E). The wheat was finely ground using a hammer mill and then mixed with 32% tap water. The resulting mixture was molded into fresh Daqu bricks measuring 34 cm × 27 cm × 6 cm. These bricks were stacked layer by layer on fermentation room shelves, where they underwent incubation for 9 d. To ensure proper aeration and temperature, the bricks were manually turned every two days during this period. On day 9, the Daqu bricks were transferred to a secondary fermentation chamber, where they were stored in tightly stacked piles for further fermentation. After a maturation period of 90 d, the fully fermented Daqu bricks were considered ready for use in the liquor fermentation process.

### 2.4. Daqu Sample Collection

Daqu samples were collected at 0, 9, and 90 d after fermentation. Triplicate samples, representing new or mature Daqu, were collected from the upper, middle, and lower layers of the fermented Daqu bricks. These samples were then pulverized and uniformly mixed. Approximately 500 g of the mixed sample was placed in sterile plastic bags and stored under two conditions: at −20 °C for the analysis of physicochemical properties and at −80 °C for microbial community structure and diversity analysis using high-throughput sequencing.

### 2.5. Quality Evaluation of Wheat Grains

The quality of grains from the three wheat cultivars grown in three cultivation environments was comprehensively evaluated. Key grain quality parameters, including moisture content (%), ash content (%), water absorption rate (%), wet gluten content (%), Zeleny sedimentation value (mL), extension (E135) (mm), farinograph quality number (FQN), maximum tensile resistance, softness, stable time (min), and volume weight (g·L^−1^), were measured using the Infratec™ 1241 Near-Infrared Grain Analyzer (FOSS Analytical, Höganäs, Sweden). Measurements were conducted at 25 °C with a scanning wavelength range of 850–1050 nm and a resolution of 2 nm [19]. Starch content was determined using the Megazyme Total Starch Kit (K-TSTA-100A) and 4–100 μg of D-glucose was determined each time as calibration. Nitrogen content was determined using a Kjeltec 8400 Analyzer (FOSS Analytical). Wheat grains were ground, sieved (0.5 mm), and 0.5 g of the sample was digested with 10 mL sulfuric acid at 420 °C for 90 min, followed by distillation with 50 mL of 40% sodium hydroxide. Calibration with 0.1 M ammonium sulfate and a blank control was performed before each analysis. The nitrogen content was then multiplied by 5.7 to calculate the wheat grain protein content. Grain hardness was measured using Brown’s Grain Hardness Tester. Additionally, seed germination potential (GP), germination rate (GR), germination index (GI), and vigor index (VI) were assessed following the germination test standard methods outlined in China’s Crop Inspection Procedures (GB/T3543.4-1995) [20].

### 2.6. Determination of Physicochemical and Enzymatic Properties of Daqu

The physicochemical and enzymatic properties of Daqu were assessed using standardized methods. The moisture content was determined using the gravimetric method by drying the samples at 105 °C for 4 h, while the acidity was measured using a pH meter [21]. The reducing sugar content was quantified following a previously established method [22], and the amino acid nitrogen content was analyzed using a colorimetric method. Enzyme activities were evaluated according to the Light Industry Standard of the People’s Republic of China (QB/T 4257-2011) [23]. Liquefying activity (U) was defined as the amount of starch liquefied in 1 g of Daqu within 1 h at 35 °C and pH 4.6, while saccharifying activity (U) represented the quantity of starch converted to glucose by 1 g of Daqu under the same conditions. Fermentation capacity (U) was measured as the amount of carbon dioxide produced by 0.5 g of fermentable sugar in Daqu over 72 h at 35 °C. Lastly, esterifying activity (U) was defined as the amount of ethyl caproate catalyzed by 50 g of Daqu at 35 °C over 7 d [4].

### 2.7. Determination of Endophytes in Wheat Grains and Microbial Communities in Daqu

The sequencing of wheat grain endophytes was conducted at Personalbio Technology Co., Ltd. (Shanghai, China), while Daqu microbial sequencing was performed at Magigene Biotechnology Co., Ltd. (Guangzhou, China). DNA extraction was performed using two different kits tailored to the specific characteristics of the wheat grain and Daqu. The Fast DNA Pro Soil-Direct Kit (MP Biomedicals, Santa Ana, CA, USA) was used for wheat grains, as it is optimized for plant tissues with high polysaccharide and polyphenol contents. For Daqu, a complex sample containing diverse microbial communities, the OMEGA Soil DNA Kit (D5625-01; Omega Bio-Tek, Norcross, GA, USA) was selected. PacBio sequencing technology was employed to sequence the 16S rRNA V5–V7 region for bacterial endophytes and the ITS2 region for fungal endophytes in wheat. For Daqu, Illumina Miseq high-throughput sequencing technology was used to sequence the 16S rRNA V1–V9 region for bacteria and the ITS2 region for fungi. The sequencing data were processed using SMRT-Link software (version 6.0) to obtain clean reads. Sequences with 97% homology were grouped into operational taxonomic units (OTUs) using USEARCH software (v10, http://www.drive5.com/usearch/, accessed on 19 February 2021). Taxonomic classification of bacterial and fungal OTUs was performed using the RDP Classifier (v2.2). Bacterial OTUs were matched against the Silva database (v132, https://www.arb-silva.de/, accessed on 8 March 2021), and fungal OTUs were classified using the Unite database (v7.2) following established protocols [24].

### 2.8. Statistical Analysis

Statistical analyses were performed to compare the quality of the wheat and Daqu using IBM-SPSS v27.0, with significance set at *p* < 0.05. The analysis of physicochemical indices and enzymatic activities was conducted using Origin 2021 [25]. Variations in the structure and diversity of dominant microbial communities in the wheat grains and Daqu across different varieties and cultivation environments were visualized using the Personalbio Genes Cloud Platform. The Mantel test was employed to examine the correlations among the wheat quality, dominant endophytes in grains, and Daqu quality. Redundancy analysis (RDA) was performed to evaluate the correlations between the dominant microbial communities in Daqu and its quality attributes [22]. Additionally, Spearman correlation coefficients were calculated to assess the correlations between dominant endophytes in wheat grains and the microbial communities in Daqu (*p* < 0.05) [26].

## 3. Results

### 3.1. Quality Analysis of Wheat Grains

The quality traits of wheat grains were significantly influenced by variety, cultivation environment, and their interactions (Table 1). Among the 18 assessed traits, FQN exhibited the highest coefficient of variation (40.66%), followed by stable time (35.22%), VI (24.03%), and softness (23.98%). In contrast, volume weight displayed the smallest variation (1.79%), followed by ash content (2.49%), moisture content (3.65%), and water absorption rate (6.64%).

Significant differences were observed across 18 quality traits among the three wheat varieties (*p* < 0.01). Based on the mean values, B4361 grains exhibited the significantly highest moisture content, maximum tensile resistance, GP, GR, GI, and VI, while displaying the significantly lowest protein content and extension. CM39 grains had the significantly highest water absorption rate, wet gluten content, Zeleny sedimentation value, FQN, grain hardness, and stable time. LNM3 grains had the significantly highest ash content and softness but the significantly lowest starch content and volume weight (*p* < 0.05).

Apart from starch content, significant (*p* < 0.05) or extremely significant (*p* < 0.01) differences were noted across 17 quality traits among the three cultivation environments. Grains cultivated in Chongzhou exhibited the highest water absorption rate, GP, GR, GI, and VI, coupled with the significantly lowest ash content. Grains from Luzhou displayed the highest softness and the lowest values for protein content, wet gluten content, extension, FQN, and grain hardness (*p* < 0.05). These findings indicate that the effect of wheat variety on grain quality was more pronounced than that of the cultivation environment.

### 3.2. Analysis of Endophyte Community Structure in Wheat Grain

A total of 4786 bacterial OTUs were identified across all wheat grains. Among them, 612 bacterial OTUs were shared among the three wheat varieties (Figure S1), while B4361 grains exhibited the highest number of unique bacterial OTUs (1517). Similarly, 589 bacterial OTUs were shared among grains cultivated in the three environments, with wheat grains from Mianzhu containing the highest number of bacterial OTUs (1506).

A total of 764 fungal OTUs were identified across all wheat grains. Among them, 78 fungal OTUs were shared among the three wheat varieties, with LNM3 grains exhibiting the highest number of unique fungal OTUs (57). Similarly, 85 fungal OTUs were shared among grains cultivated in the three environments, while wheat grains from Luzhou had the highest number of fungal OTUs (94).

**Figure 1:**
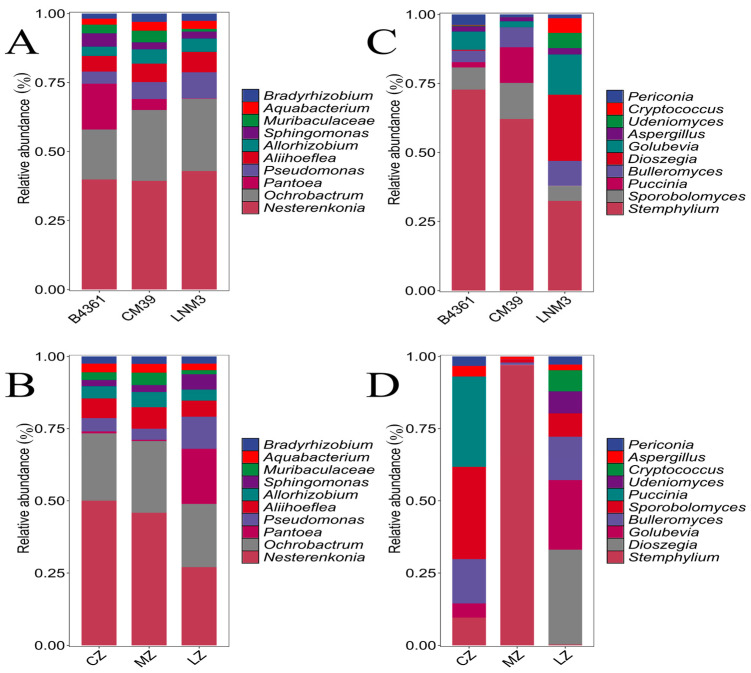
The relative abundance of dominant endophytes in wheat grains. Note: The relative abundance of endophytic bacteria in three varieties (**A**) and three cultivation environments (**B**) at the genus level; the relative abundance of endophytic fungi at the genus level in three varieties (**C**) and three cultivation environments (**D**). CZ, MZ and LZ represent Chongzhou, Mianzhu and Luzhou, respectively (the same below).

At the genus level, the dominant bacterial genera across all wheat grains were *Nesterenkonia* and *Ochrobactrum*. In addition, *Pantoea* exhibited significantly higher relative abundance in B4361 grains (16.57%) and grains cultivated in Luzhou (19.05%) (*p* < 0.05; Figure 1A,B). For fungi, the dominant genus in grains of all three wheat varieties was *Stemphylium* (Figure 1C). Notably, *Sporobolomyces* (13.10%) and *Puccinia* (12.96%) were more abundant in CM39 grains, whereas *Dioszegia* (23.98%) and *Golubevia* (14.54%) showed significantly higher relative abundance in LNM3 grains compared to the other two varieties (*p* < 0.05). The dominant fungal genera varied across cultivation environments (Figure 1D). In grains from Chongzhou, *Sporobolomyces* (31.94%) and *Puccinia* (31.21%) were predominant. In Mianzhu grains, *Stemphylium* (97.03%) dominated, while in Luzhou grains, *Dioszegia* (32.78%) and *Golubevia* (24.09%) were the most abundant. These results suggest that the influence of wheat species and cultivation environment on endophytic fungal communities is greater than on bacterial communities, with the cultivation environment having the most pronounced impact.

### 3.3. Diversity Analysis of Endophyte Community in Wheat Grains

**Figure 2:**
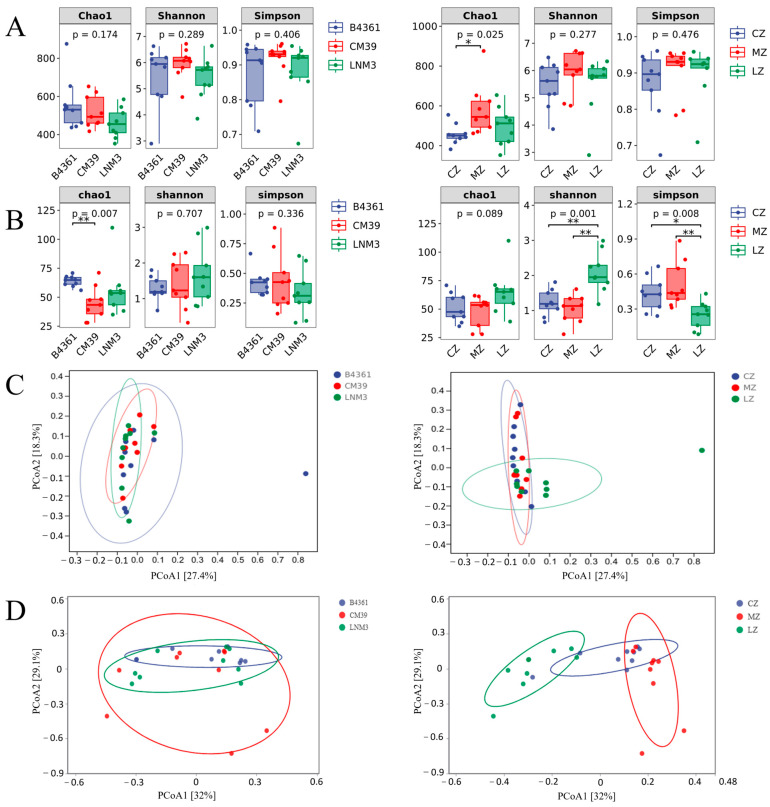
Analysis of α and β diversity of endophyte in wheat grains. Note: α diversity analysis of endophytic bacteria in three varieties and cultivation environments (**A**) at the genus level; α diversity of endophytic fungi at the genus level was analyzed in three varieties and cultivation environments (**B**). * and ** indicated that the two groups of connections were significantly correlated at the 0.05 and 0.01 levels, respectively. β diversity analysis (PCoA) of endophytic bacteria in three varieties and cultivation environments (**C**) at the genus level; β diversity analysis (PCoA) of endophytic fungi at the genus level in three varieties and cultivation environments (**D**). Chao1 index was positively correlated with microbial richness, Shannon index was positively correlated with microbial diversity, and Simpson index was negatively correlated with microbial diversity. Lines and dots of the same color correspond to the same variety and cultivation environment.

The alpha (α) diversity analysis of the endophyte community in wheat grains (Figure 2A,B) revealed that the richness of endophytic bacteria, measured by the Chao1 index, varied significantly (*p* < 0.05) among the three cultivation environments, while the richness of endophytic fungi showed highly significant differences (*p* < 0.01) among the three wheat varieties. Additionally, the diversity of endophytic fungi, assessed using the Shannon index, displayed significant differences (*p* < 0.01) among cultivation environments. Among the varieties, CM39 grains exhibited the highest richness and diversity of endophytic bacteria, while B4361 grains showed the greatest richness of endophytic fungi. Regarding the cultivation environments, grains grown in Mianzhu demonstrated the highest richness and diversity of endophytic bacteria, whereas grains cultivated in Luzhou displayed the highest richness and diversity of endophytic fungi.

To assess the similarity or dissimilarity of endophyte community composition in wheat grains, principal coordinate analysis (PCoA) based on Bray–Curtis dissimilarity was conducted (Figure 2C,D). The confidence intervals of the three wheat varieties were closely linked, indicating a high degree of similarity in community structure across varieties. However, from an environmental perspective, the bacterial community composition in grains grown in Chongzhou showed high consistency with that of those grown in Mianzhu but differed significantly from that of grains cultivated in Luzhou. For fungal communities, significant differences were observed among the confidence groups of the three cultivation environments. These findings underscore that compared to variety, environmental factors exert a greater influence on the diversity of endophytic communities in wheat grains. Notably, the impact of the environment is the most pronounced on fungal community composition.

### 3.4. Dynamics of Physicochemical Properties of Daqu During Fermentation

**Figure 3:**
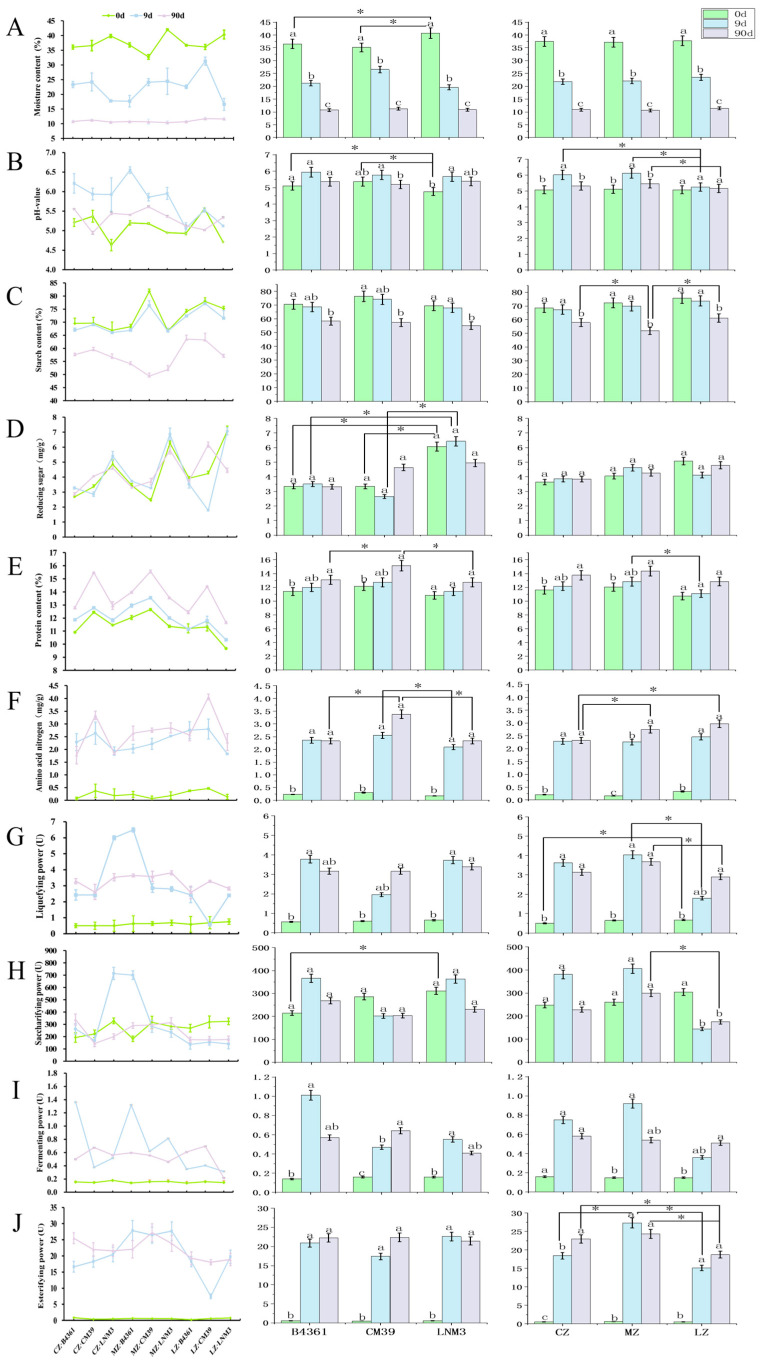
The performance of physicochemical and enzyme activity indexes during the fermentation process of Daqu from different sources of wheat grains. Note: (**A**) Moisture content; (**B**) pH value; (**C**) Starch content; (**D**) Reducing sugar; (**E**) Protein content; (**F**) Amino acid nitrogen; (**G**) Liquefying activity; (**H**) Saccharifying activity; (**I**) Fermentation capacity; (**J**) Esterifying activity. The line chart (left diagram) shows the change trend of all samples; the bar chart (right picture) is grouped according to variety and cultivation environment, indicating the average value of the group. The same lowercase letters on the bar chart indicate that the difference between distinct varieties or environments did not reach a significant level of 0.05; * indicate that the two groups of connections are significantly correlated at the 0.05 levels, respectively. The nine samples in the left diagram are named after the cultivation environment + variety, for example: CZ·B4361 represents B4361 wheat cultivated in Chongzhou.

The physicochemical properties of Daqu exhibited distinct trends throughout the fermentation process. Moisture and starch content both showed a decreasing trend (Figure 3), with moisture content varying significantly across different fermentation periods (*p* < 0.05). Starch content demonstrated a significant decline between days 9 and 90 of fermentation (*p* < 0.05). Protein content exhibited a gradual upward trend over the fermentation period. Amino acid nitrogen levels increased significantly during the early stages of fermentation (0–9 d; *p* < 0.05), with a reduced rate of increase observed during the post-ripening storage phase. The pH value of Daqu increased significantly by day 9 of fermentation, transitioning from an acidic to a neutral state. Subsequently, the pH value gradually declined, returning to acidic during the later stages of fermentation.

Varietal differences significantly influenced the physicochemical properties of Daqu during fermentation. On day 0, LNM3 Daqu exhibited the significantly highest moisture content and significantly lowest pH value compared to the Daqu prepared from the grains of the other two cultivars. Additionally, it exhibited significantly higher reducing sugar content on days 0 and 9 (*p* < 0.05). By day 90, CM39 Daqu showed significantly higher protein content and amino acid nitrogen levels than Daqu prepared from the other grain varieties (*p* < 0.05). Cultivation environment also impacted the physicochemical properties of Daqu. On day 9, Daqu prepared from grains cultivated in Luzhou had a significantly lower pH value compared to that prepared from grains from the other two environments (*p* < 0.05). By day 90, Daqu prepared from Mianzhu grains exhibited the lowest starch content, while that prepared from Chongzhou grains showed significantly lower amino acid nitrogen levels than that prepared from grains cultivated in the other two environments (*p* < 0.05). Overall, varietal differences had a greater impact on the physicochemical properties of Daqu than that the cultivation environment.

### 3.5. Dynamic Changes in Daqu Enzyme Activity During Fermentation

The enzyme activity of Daqu exhibited notable dynamic trends during fermentation, with liquefying activity, fermentation capacity, and esterifying activity all increasing significantly during the initial fermentation phase (0–9 d; *p* < 0.05) (Figure 3). In terms of variety, the saccharifying activity of LNM3 Daqu was significantly higher than that of B4361 Daqu on day 0 (*p* < 0.05). In terms of environmental influence, the liquefying activity of Mianzhu Daqu was significantly higher than that of Luzhou Daqu on days 9 and 90 (*p* < 0.05). Similarly, the saccharifying activity of Mianzhu Daqu surpassed that of Luzhou Daqu on day 90 (*p* < 0.05). On day 9, the esterifying activity of Mianzhu Daqu was significantly higher than that of Daqu prepared from grains cultivated in the other two environments (*p* < 0.05), while by day 90, the esterifying activity of Chongzhou and Mianzhu Daqu was significantly higher than that of Luzhou Daqu (*p* < 0.05). Overall, the cultivation environment had a greater impact on Daqu enzyme activity than wheat variety, with Mianzhu Daqu consistently demonstrating superior enzyme activity across multiple parameters.

### 3.6. Analysis of Microbial Community Structure of Daqu

A total of 4786 bacterial OTUs were identified across all Daqu samples. Among the three fermentation stages, 545 bacterial OTUs were shared (Figure S2). On day 90, Daqu samples exhibited the highest number of unique OTUs, with 1076 bacterial OTUs. The shared OTUs for Daqu bacteria across varieties and environments accounted for 97.64% and 98.14% of the total bacterial OTUs, respectively.

Similarly, 764 fungal OTUs were identified across all Daqu samples, with 116 fungal OTUs shared among the three fermentation stages. On day 90, Daqu samples exhibited the highest number of unique OTUs, with 92 fungal OTUs. The shared OTUs for Daqu fungi across varieties and environments accounted for 99.95% of the total fungal OTUs, demonstrating a high degree of similarity. These results indicate that microorganisms in Daqu from different wheat varieties and cultivation environments tend to converge during fermentation.

**Figure 4:**
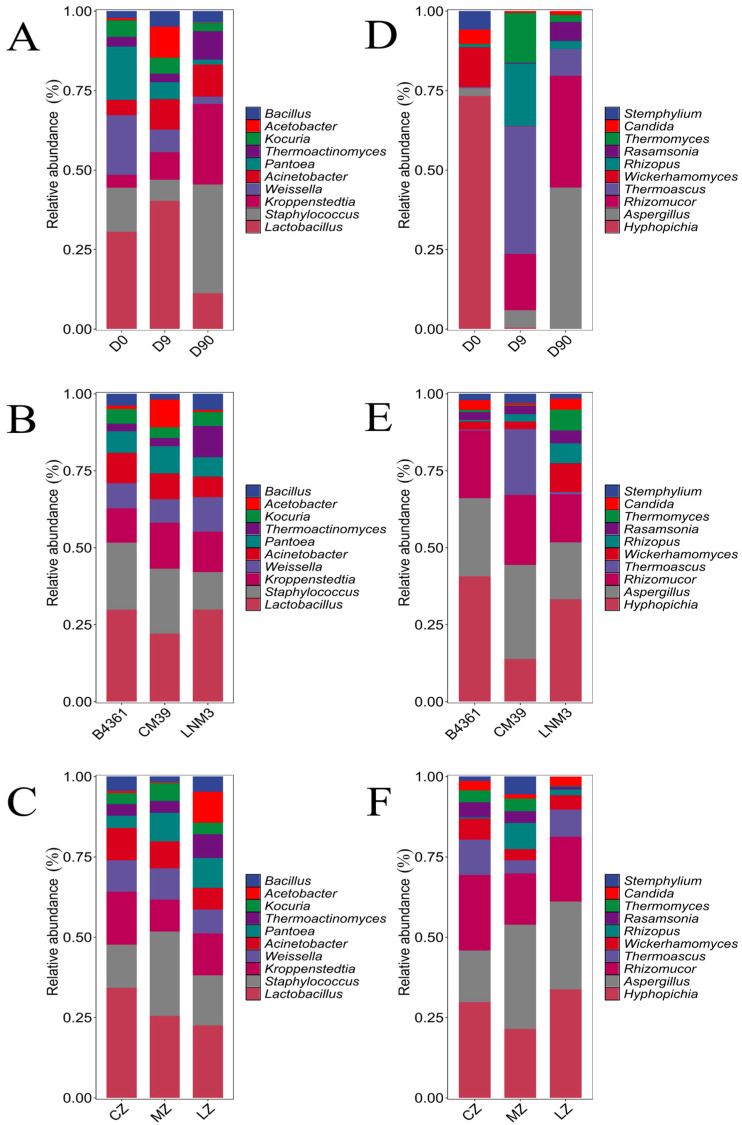
Relative abundance of dominant bacteria in Daqu. Note: The relative abundance of Daqu bacteria at the genus level at three fermentation times (**A**), three culture environments (**B**) and three varieties (**C**); the relative abundance of Daqu fungi at the genus level at three fermentation times (**D**), three culture environments (**E**) and three varieties (**F**). D0, D9, and D90 represent the 0th, 9th, and 90th day of Daqu fermentation, respectively (the same below).

At the genus level, the dominant bacterial genera varied across fermentation stages (Figure 4A). On day 0, the dominant genera were *Lactobacillus* (as defined by RDP Classifier v2.2) (30.60%), *Weissella* (18.81%), *Pantoea* (16.71%), and *Staphylococcus* (13.85%). By day 9, *Lactobacillus* (40.31%), remained dominant, with *Acetobacter* (9.78%) also showing a high relative abundance, significantly greater than in the other fermentation stages (*p* < 0.05). On day 90, the dominant bacterial genera shifted to *Staphylococcus* (34.25%) and *Kroppenstedtia* (25.29%), with *Thermoactinomyces* (8.96%) also displaying significantly higher relative abundance than at other fermentation stages (*p* < 0.05). Across different wheat varieties and cultivation environments, the dominant bacterial genera—*Lactobacillus*, *Staphylococcus* and *Kroppenstedtia*—remained consistent (Figure 4B,C).

The dominant fungal genera in Daqu varied across fermentation stages (Figure 4D). On day 0, *Hyphopichia* (73.30%), *Wickerhamomyces* (12.6%), and *Stempylium* (5.85%) were the most abundant, with significantly higher relative abundances than at the other two stages (*p* < 0.05). By day 9, the dominant fungal genera shifted to *Thermoascus* (40.14%), *Rhizopus* (19.59%), *Rhizomucor* (17.72%), and *Thermomyces* (15.71%). On day 90, the dominant fungal genera were *Aspergillus* (44.50%) and *Rhizomucor* (35.14%). In terms of variety (Figure 4E), the dominant fungal genera in Daqu prepared from B4361 and LNM3 grains were consistent: *Hyphopichi*, *Aspergillus*, and *Rhizomucor*. For CM39, the dominant genera included *Aspergillus* (30.58%), *Rhizomucor* (22.80%), and *Thermoascus* (21.33%). Across different cultivation environments, the dominant fungal genera remained the same: *Hyphopichi*, *Aspergillus*, and *Rhizomucor* (Figure 4F). These findings suggest that the effect of fermentation time on the Daqu microbial community was greater than that of variety and environment.

### 3.7. Diversity Analysis of Daqu Microbial Community

**Figure 5:**
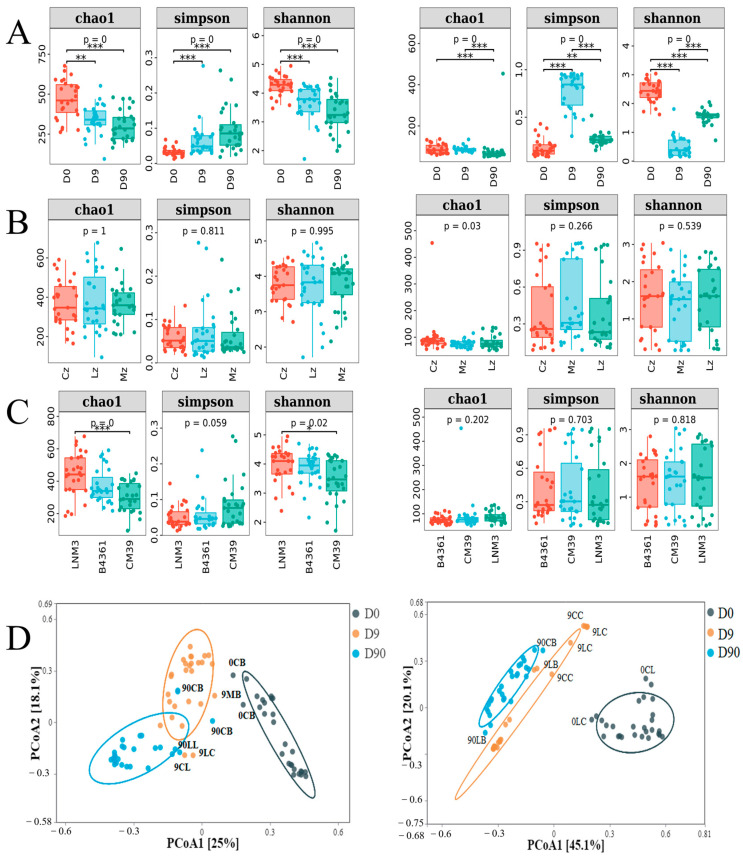
The α and β diversity of the Daqu microbial community. Note: The α diversity of Daqu bacteria and fungi at three fermentation times (**A**), three culture environments (**B**) and three varieties (**C**); *, ** and *** indicated that the two groups of connections were significantly correlated at the 0.05, 0.01, 0.001 levels, respectively. β diversity analysis (PCoA) of Daqu bacteria and fungi at the genus level was analyzed at three fermentation times (**D**). Lines and points of the same color correspond to the same fermentation time.

The alpha (α) diversity analysis of the Daqu microbial community revealed significant trends during fermentation (Figure 5A–C). The bacterial richness (Chao1 index) and diversity (Shannon index) decreased significantly over time (*p* < 0.01). Similarly, fungal richness (Chao1 index) exhibited a significant decline (*p* < 0.01), and fungal diversity (Shannon index) showed a distinct pattern: a significant reduction by day 9, followed by a partial recovery, reaching approximately half its initial level by day 90 (*p* < 0.01) (Figure 5A). Significant differences were observed in bacterial community richness (Chao1) (*p* < 0.01) and diversity (Shannon) (*p* < 0.05) among Daqu derived from different wheat varieties, with LNM3 Daqu demonstrating the highest bacterial richness and diversity (Figure 5C). Additionally, fungal community richness (Chao1) varied significantly among Daqu produced in different environments (*p* < 0.05) (Figure 5B). Beta (β) diversity analysis showed that bacterial and fungal communities in the same fermentation period clustered distinctly. These findings highlight significant differences in microbial community diversity across fermentation stages.

### 3.8. Mantel Correlation Analysis of Grain Endophytes with Wheat Raw Grain and Daqu Quality

**Figure 6:**
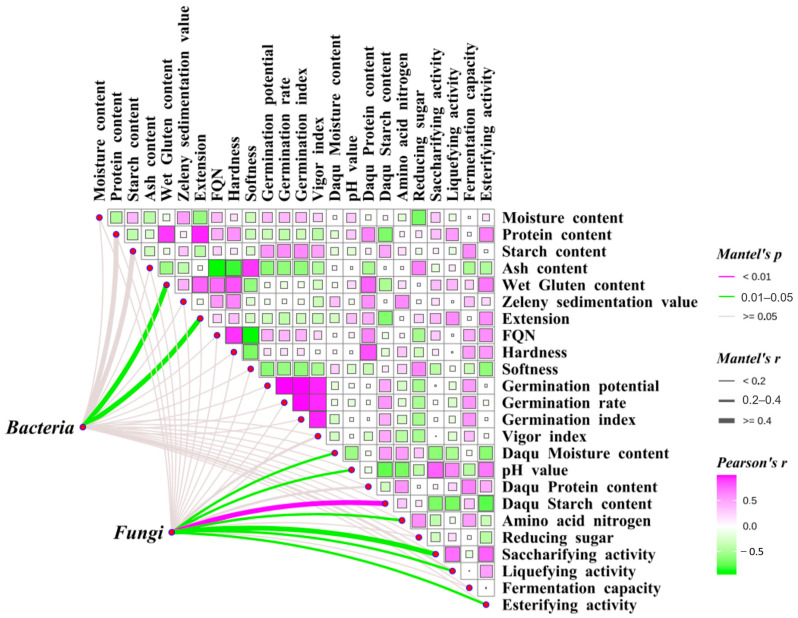
Mantel correlation analysis of wheat raw grain quality with grain endophyte and Daqu quality. Note: A purple line represents a highly significant correlation between the connection points (*p* < 0.01), while a green line represents a significant correlation between the connection points (*p* < 0.05). A purple square represents a significant positive correlation between two quality indicators, while a green square represents a significant negative correlation between the two quality indicators (*p* < 0.05). The size of the squares and color intensity represent the strength of association between the connected metrics.

Mantel correlation analysis was performed to investigate the relationships among wheat endophytes, wheat quality, and Daqu quality (Figure 6). The results revealed significant positive correlations between wheat protein content, wet gluten content, FQN, and grain hardness and the protein content and esterifying activity of Daqu (*p* < 0.05). Conversely, the protein content, wet gluten content, and extension of wheat grains were significantly negatively correlated with the starch content of Daqu (*p* < 0.05). Additionally, the starch content of wheat grains was significantly positively correlated with the fermentation capacity of Daqu (*p* < 0.05). The abundance of dominant endophytic bacteria in wheat grains showed significant positive correlations with wet gluten content (r = 0.61) and extension (r = 0.69) (*p* < 0.05). Similarly, the abundance of dominant endophytic fungi in wheat grains exhibited significant positive correlations with various Daqu quality traits, including starch content (r = 0.45; *p* < 0.01), moisture content (r = 0.26), pH (r = 0.28), amino acid nitrogen levels (r = 0.26), saccharifying activity (r = 0.41), liquefying activity (r = 0.31), and esterifying activity (r = 0.30; *p* < 0.05).

### 3.9. Correlation Between Daqu Quality and Microbial Community via RDA

**Figure 7:**
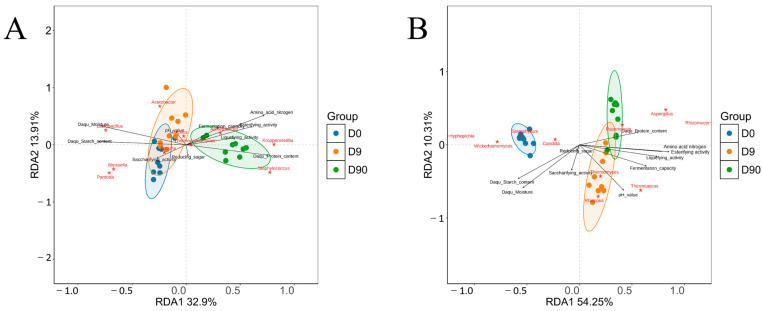
RDA of the correlation between Daqu quality and Daqu microbial community. Note: RDA analysis of bacterial community and quality indicators of Daqu (**A**); RDA analysis of fungal community and quality indicators of Daqu (**B**). Lines and points of the same color correspond to the same fermentation time.

RDA was performed to explore the relationships between 10 Daqu quality indicators and the dominant genera of Daqu microbial community (Figure 7). The analysis revealed that at different fermentation stages, distinct bacterial genera were associated with specific Daqu quality traits. On day 0, moisture content, starch content, and saccharifying activity of Daqu were positively correlated with the abundance of bacterial genera such as *Lactobacillus*, *Pantoea*, *Weissella*, and *Kocuria*. By day 9, the pH value of Daqu showed a positive correlation with the abundance of *Acetobacter* and *Bacillus*. On day 90, protein content, amino acid nitrogen content, liquefying activity, fermentation capacity, and esterifying activity were positively correlated with the abundance of *Kroppenstedtia*, *Staphylococcus*, *Acinetobacter*, and *Thermoactinomyces*. Among the quality indicators, moisture, starch, protein, and amino acid nitrogen contents of Daqu exhibited the most substantial impact in shaping the abundance and differentiation of bacterial community.

In terms of the fungal community, on day 0, moisture content, starch content, reducing sugar, and saccharifying activity of Daqu were positively correlated with the abundance of *Hyphopichia*, *Wickerhamomyces*, *Candida*, and *Stephylium*. By day 9, the pH value of Daqu showed a positive correlation with *Thermomyces*, *Thermoascus*, and *Rhizopus* abundance. On day 90, protein content, amino acid nitrogen content, liquefying activity, fermentation capacity, and esterifying activity of Daqu were positively correlated with the abundance of *Rhizomucor*, *Rasamsonia*, and *Aspergillus*. Among the quality indicators, moisture content, pH value, starch content, amino acid nitrogen content, and esterifying activity had the most substantial impact in shaping the fungal community structure in Daqu. These results underscore the intricate relationship between the microbial community structure and evolving quality characteristics of Daqu during fermentation.

### 3.10. Correlation Analysis of Microbial Communities in Daqu

**Figure 8:**
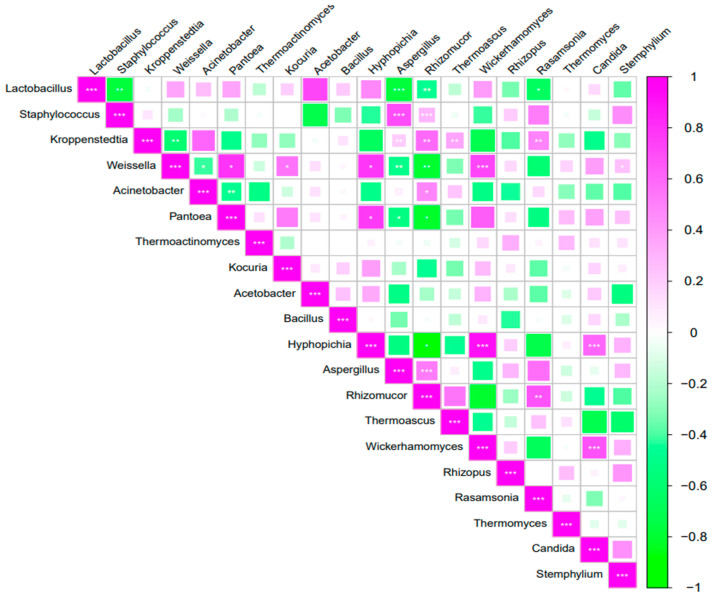
Correlation analysis of microbial communities in Daqu. Note: * represents *p* ≤ 0.05; ** represents *p* ≤ 0.01. ***represents *p* ≤ 0.001. The size of the squares and color intensity represent the strength of association between the connected metrics.

Spearman correlation analysis was conducted on the top 20 bacterial and fungal genera with the highest relative abundance (Figure 8). Among bacterial genera, significant negative correlations emerged between *Lactobacillus* and *Staphylococcus* (*p* < 0.01), as well as between *Kroppenstedtia* and *Weissella* (*p* < 0.01). *Weissella* demonstrated particularly complex associations, showing negative correlations with *Acinetobacter* but positive relationships with both *Pantoea* and *Kocuria* (*p* < 0.05). Notably, the abundances of *Acinetobacter* and *Pantoea* exhibited a strong negative covariation (*p* < 0.01). Fungal interactions contrasted with the bacterial patterns, displaying predominantly positive associations. The abundance of *Hyphopichia* showed strong positive correlations with both *Wickerhamomyces* and *Candida* (*p* < 0.01), while *Aspergillus* was positively correlated with *Rhizomucor* (*p* < 0.01). The *Rhizomucor*–*Rasamsonia* pair maintained a significant positive correlation (*p* < 0.01), as did *Wickerhamomyces* and *Candida* (*p* < 0.01), suggesting extensive fungal synergy during fermentation.

Cross-domain analysis revealed intricate bacterial–fungal interactions. *Aspergillus* (fungi) and *Rhizomucor* (fungi) abundances were significantly negatively correlated with those of *Lactobacillus* (*p* < 0.01), *Weissella* (*p* < 0.01), and *Pantoea* (*p* < 0.05), but showed positive associations with *Staphylococcus* and *Kroppenstedtia* (*p* < 0.01). Additionally, the abundance of *Hyphopichia* (fungi) displayed positive correlations with *Weissella* and *Pantoea* (*p* < 0.05), while *Thermoascus* (fungi) and *Rasamsonia* (fungi) correlated positively with *Kroppenstedtia* (*p* < 0.01). A particularly strong positive association emerged between *Wickerhamomyces* and *Weissella* (*p* < 0.01). These complex cross-kingdom relationships highlight the sophisticated microbial network underlying Daqu fermentation.

### 3.11. Correlation Assessment Between Wheat Grain Endophytes and Daqu Microbial Community

**Figure 9:**
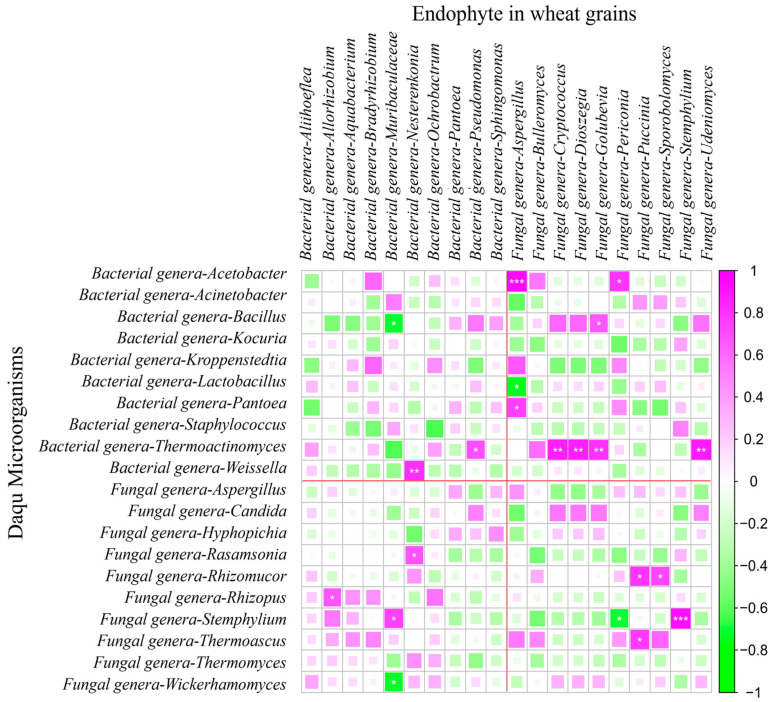
Correlation analysis between wheat grain endophytes and Daqu microbial community. Note: * represents *p* ≤ 0.05; ** represents *p* ≤ 0.01. *** represents *p* ≤ 0.001. Daqu microorganisms on the left and wheat grain endophytes on the top. The size of the squares and color intensity represent the strength of association between the connected metrics.

Spearman correlation analysis revealed significant associations between the top 20 dominant endophytic genera in wheat grains and Daqu microbiota (Figure 9).

For bacterial correlations of wheat grain, four wheat endophytic genera displayed notable correlations with Daqu communities. *Allorhizobium* abundance positively correlated with *Rhizopus* (fungi) (r = 0.67) (*p* < 0.05), while *Muribaculaceae* showed pronounced covariation with *Stemphylium* (fungi) (r = 0.75) (*p* < 0.05). *Nesterenkonia* exhibited dual associations, linking to both *Weissella* (bacteria) (r = 0.82) (*p* < 0.01) and *Rasamsonia* (fungi) (r = 0.68) (*p* < 0.05). *Pseudomonas* abundance positively correlated with *Thermoactinomyces* (bacteria) (r = 0.69) (*p* < 0.05).

Fungal interactions demonstrated broader cross-domain effects, with nine wheat endophytic genera influencing the Daqu microbiota. *Aspergillus* showed exceptionally strong associations with *Acetobacter* (bacteria) (r = 0.94) (*p* < 0.01) and *Pantoea* (bacteria) (r = 0.76) (*p* < 0.05). Moreover, the abundances of *Cryptococcus*, *Dioszegia*, *Golubevia* and *Udeniomyces* shared robust correlations with *Thermoactinomyces* (bacteria) (*p* < 0.01). *Periconia* abundance exhibited associations with *Acetobacter* (bacteria) (r = 0.79) (*p* < 0.05). *Puccinia* correlated with both *Rhizomucor* (fungi) (r = 0.79) (*p* < 0.05) and *Thermoascus* (fungi) (r = 0.77) (*p* < 0.05). *Sporobolomyces* correlated with *Rhizomucor* (fungi) (r = 0.72) (*p* < 0.05), and intriguingly, *Stemphylium* displayed self-correlation (r = 0.93) (*p* < 0.01). In conclusion, the abundance of endophytic fungal genera in wheat seeds has a greater impact on the microbial community abundance of Daqu, particularly the abundance of *Thermoactinomyces* (bacteria) in Daqu.

## 4. Discussion

MT-Daqu, prepared from wheat through solid-state fermentation, serves as an essential saccharification starter in liquor brewing [27]. The quality of wheat, the primary raw material for Daqu, is influenced by wheat varieties, cultivation environments, and their interactions [28]. In this study, these factors significantly affected key quality traits of wheat. For example, CM39 grains and all grains cultivated in Mianzhu had the highest protein content, while B4361 grains and all grains cultivated in Luzhou exhibited the highest starch content, providing abundant carbon and nitrogen sources for fermentation microorganisms. These characteristics likely promote the growth and activity of Daqu microorganisms [8]. Additionally, B4361 grains and all grains cultivated in Chongzhou exhibited the highest VI value. Pre-harvest sprouting has been shown to significantly impact the baking/cooking qualities of wheat-based products, such as bread, steamed bread, and noodles [29], and a high germination rate has been confirmed to play a significant role in brewing beer. However, their impact on Daqu fermentation remains to be further investigated.

The influence of wheat on the Daqu microbial community has long been a central focus of the industry. With the advent of traceability analysis, attention has gradually shifted from wheat quality to the role of wheat endophytes. Previous studies employing PacBio sequencing technology identified *Staphylococcus*, *Pantoea*, *Alternaria*, and *Mycosphaerella* as dominant genera in wheat-derived flora [2]. In this study, the same sequencing approach revealed *Nesterenkonia*, *Ochrobactrum*, and *Stemphylium* as the dominant endophytic genera in wheat.

The composition of endophytes is influenced by various factors, including the environment, host variety, and their interactions [30]. Endophytes exhibit characteristics such as a wide distribution range, significant variation in number, diverse species, and regional specificity [31]. Therefore, the dominant endophytes vary significantly across different wheat varieties and cultivation environments. This study further revealed associations between wheat grain composition and endophytes: protein content was related to the abundance of endophytic bacteria, while starch content was associated with endophytic fungi abundance. CM39 grains and all grains cultivated in Mianzhu exhibited the highest protein content, along with the highest richness and diversity of endophytic bacteria. Similarly, B4361 grains cultivated in Luzhou displayed the highest starch content, which corresponded to the highest richness of endophytic fungi. In addition, a Mantel test identified a significant positive correlation between the abundance of dominant endophytic fungi in wheat grains and starch content in Daqu, suggesting a potential link to the long-term mutualism between endophytes and the nutritional quality of their host plants.

The physicochemical properties and enzyme activity index of Daqu play a critical role in determining its quality and application [32]. Variations in Daqu properties are influenced by the wheat grains used, which differ by variety and cultivation environment. The low moisture content of Daqu may result from the loose microporous structure of Daqu bricks, attributed to the high nitrogen content in the raw materials [19]. Our findings that CM39 Daqu exhibited low moisture and high protein content, align with these previous results. The significantly higher acidity observed in LNM3 Daqu compared to that prepared from other wheat varieties was linked to the abundance of dominant acid-producing bacteria, including *Weissella* and *Lactobacillus*. Additionally, *Aspergillus*, known for its ability to secrete enzymes such as amylase, protease, and glucoamylase, contributes significantly to Daqu functionality [33]. Daqu prepared from Mianzhu grains displayed superior liquefying activity, saccharifying activity, fermentation capacity, and esterifying activity compared to that prepared using grains from the other two environments. This aligns with the microbial community analysis, which revealed a higher abundance of *Aspergillus* in Mianzhu Daqu. Consequently, multiple indices of Mianzhu Daqu quality surpassed those of its counterparts, highlighting the synergistic relationship between microbial abundance and Daqu properties.

From the perspective of microbial community succession, distinct patterns of microbial abundance were observed throughout the fermentation process of Daqu. During the early stage, *Weissella*, *Pantoea*, and *Lactobacillus* were the dominant genera, with their abundance exhibiting a gradual decline as fermentation progressed, consistent with previous findings [34]. *Weissella*, an acid-producing bacterium, exhibited significantly higher richness on day 0, correlating with the lower pH value of Daqu at this stage. *Pantoea*, primarily sourced from wheat [35], was also abundant in the early fermentation phase, but its richness significantly decreased thereafter, aligning with prior reported results [36]. Conversely, the abundance of *Staphylococcus* and *Kroppenstedtia* significantly increased from day 9 to 90, serving as an indicator of Daqu maturation [37]. The antagonistic interactions among *Lactobacillus*, *Staphylococcus* and *Kroppenstedtia* were evident. Correlation analysis revealed a significant negative correlation between the abundance of *Lactobacillus* and *Staphylococcus*, indicating possible competition for limited growth substrates, and the decline of *Lactobacillus* abundance appeared to facilitate the growth of *Kroppenstedtia* [38]. In the fungal community, *Hyphopichia* was found in large quantities on day 0, consistent with its primary source being the fermentation environment (including the floor and tools) [33]. The raw wheat grain-derived fungus *Stephylium* also participated in the initial fermentation, showing relatively high abundance on day 0 but disappearing by day 9. During the heating period, *Rhizomucor* emerged as a dominant fungal genus on day 9, contributing essential enzymes and amino acid nitrogen for liquor fermentation [39]. Similarly, *Thermoascus* and *Thermomyces* fungi were prominent on day 9, thriving under elevated temperatures due to their high-temperature tolerance. These fungi became predominant as the rising temperature inhibited the growth of less heat-resistant microorganisms.

Previous studies have highlighted the critical role of temperature, moisture, and pH value in shaping the succession of the microbial community in Daqu [38]. Building on these findings, this study identified moisture and starch contents as the primary factors influencing the microbial community abundance on day 0 of fermentation. By day 9, pH value emerged as the dominant factor driving microbial dynamics, while amino acid nitrogen level became the key influencing factor by day 90. These results suggest that starch content and amino acid nitrogen level play vital roles in the microbial succession during Daqu fermentation.

The correlation analysis in this study revealed significant associations between certain wheat endophytes—such as *Nesterenkonia*, *Aspergillus*, *Cryptococcus*, *Dioszegia*, *Golubevia*, *Udeniomyces* and *Stemphylium*—and the microbial community abundance of Daqu. Among these, *Aspergillus* has been extensively reported as a critical functional fungal genus in wheat-derived microbiota. It actively participates in various metabolic pathways, particularly during the early stages of fermentation [2]. The other endophytes identified may also contribute significantly to Daqu’s microbial dynamics. These organisms potentially drive microbial evolution through secondary metabolism, enriching the functional diversity of the Daqu flora [40,41]. However, their specific roles and mechanisms of action remain unclear and warrant further investigation to fully elucidate their contributions to the fermentation process.

## 5. Conclusions

This study provides a comprehensive understanding of the differences and correlations among wheat quality, wheat endophytes, Daqu quality, and Daqu microbial communities across different varieties and cultivation environments. The findings revealed that wheat variety has a greater influence on the wheat grain quality and physicochemical properties of Daqu than the cultivation environment. However, the environment significantly impacts the richness and diversity of wheat endophytes and the enzyme activity of Daqu. CM39 wheat grains grown in Mianzhu, characterized by a high protein content and elevated enzyme activity, were consistently found to be more suitable for Daqu production. Beyond moisture content and pH, starch and amino acid nitrogen contents also play critical roles in shaping the succession of Daqu microbial communities. Notably, *Pantoea*, *Aspergillus* and *Stephylium* emerged as dominant endophytes in wheat and key microorganisms in Daqu. This study identified *Nesterenkonia*, *Aspergillus*, *Cryptococcus*, *Dioszegia*, *Golubevia*, *Udeniomyces* and *Stemphylium* as dominant wheat-derived bacterial genera associated with the abundance of microorganisms in MT-Daqu. The roles of these specific microbial genera can be directly leveraged to optimize Daqu production. For example, wheat varieties enriched with key microbial taxa can be selected, and cultivation environments can be optimized to promote the growth of beneficial endophytes. Additionally, monitoring the relative abundance of key microbial genera (e.g., *Aspergillus* and *Stemphylium*) during fermentation may serve as a biomarker for assessing Daqu quality and predicting fermentation outcomes. However, in this study, no significant differences were observed in the dominant bacterial genera across the three wheat varieties and cultivation environments, making it unclear whether these genera originated from the grains themselves or the external environment. Future research should focus on traceability analyses or multi-environment, multi genotype experiments to identify optimal grains and cultivation environments for Daqu production. This study revealed the influence of wheat derived microorganisms on the quality and microbial community of Daqu, providing a reference for the selection of Daqu raw materials and the breeding of specialized brewing varieties.

## Figures and Tables

**Table 1:** Analysis of basic parameters and variance of grain quality and seed vigor of wheat raw grains quality characteristics.

			Variety			Cultivated Environment			Average	Variance Analysis (F Value)		
			B4361	CM39	LNM3	Chongzhou	Mianzhu	Luzhou		Variety	Environment	Variety × Environment
Grain quality	Moisture content/%	Average	10.60 a	10.19 b	9.90 c	10.12 a	10.26 a	10.32 a	10.23	48.11 **	3.80 *	10.04 **
		CV/%	0.83	2.14	2.64	4.28	3.69	1.41	3.65			
	Protein content/%	Average	11.44 b	13.32 a	12.87 a	13.13 a	13.32 a	11.20 b	12.55	43.73 **	62.23 **	2.05
		CV/%	10.54	6.46	6.72	3.81	6.72	9.25	10.58			
	Starch content/%	Average	71.33 a	68.33 a	63.05 b	67.60 a	66.48 a	68.63 a	67.57	8.11 **	0.53	3.70 *
		CV/%	9.76	4.31	6.39	7.31	4.96	12.27	8.90			
	Ash content/%	Average	0.43 b	0.43 b	0.45 a	0.43 b	0.44 a	0.44 a	0.44	18.00 **	21.38 **	2.81
		CV/%	2.21	1.92	0.93	2.05	2.00	0.35	2.49			
	Water absorption rate/%	Average	57.36 b	60.07 a	52.25 c	58.30 a	56.83 ab	54.54 b	56.56	259.58 **	59.02 **	10.28 **
		CV/%	3.81	3.62	0.72	6.91	6.45	3.82	6.64			
	Wet gluten content/%	Average	28.23 b	31.91 a	28.91 b	31.06 a	30.87 a	27.11 b	29.68	34.39 **	44.41 **	4.21 *
		CV/%	9.72	4.74	4.89	4.47	5.65	7.92	9.02			
	Zeleny sedimentation value/mL	Average	32.9 b	37.58 a	27.01 c	29.77 a	34.56 a	33.17 a	32.50	96.53 **	20.87 **	11.46 **
		CV/%	5.32	7.08	14.49	19.70	11.62	13.12	16.55			
	Extension (E135)/mm	Average	133.77 b	150.65 a	154.70 a	154.18 a	154.13 a	130.81 b	146.38	54.82 **	80.85 **	5.30 **
		CV/%	10.91	4.77	7.82	3.02	7.31	9.22	10.42			
	Farinograph quality number (FQN)	Average	57.06 a	66.06 a	30.71 b	66.03 a	53.18 a	34.62 b	51.28	258.99 **	191.26 **	20.56 **
		CV/%	34.73	20.42	18.14	30.52	31.40	28.51	40.66			
	Hardness	Average	56.64 b	65.46 a	51.01 c	61.35 a	59.27 a	52.49 b	57.71	188.05 **	76.01 **	6.73 **
		CV/%	8.70	7.44	3.08	11.59	11.18	8.36	12.72			
	Maximum tensile resistance	Average	496.59 a	463.91 b	379.22 c	455.46 a	457.41 a	426.84 a	446.57	262.34 **	20.94 **	25.58 **
		CV/%	8.21	3.49	2.53	16.14	10.81	7.11	12.67			
	Softness	Average	119.67 b	131.87 b	161.33 a	105.01 c	138.15 b	169.71 a	137.62	401.46 **	916.26 **	51.72 **
		CV/%	32.23	17.29	11.13	26.41	12.36	5.68	23.98			
	Stable time/min	Average	3.70 a	4.51 a	2.59 b	4.08 a	3.84 a	2.88 a	3.60	18.75 **	8.15 **	6.51 **
		CV/%	30.27	18.30	15.02	34.06	25.74	16.50	35.22			
	Volume weight/(g·L^−1^)	Average	818.75 a	811.61 a	794.42 b	813.82 a	801.57 a	809.38 a	808.26	20.62 **	5.07 *	3.33 *
		CV/%	0.94	0.37	1.32	1.62	1.91	0.31	1.79			
Seed vigor	Germination potential%	Average	92.67 a	79.33 b	73.78 c	90.67 a	75.78 b	79.33 b	81.93	59.64 **	38.26 **	7.23 **
		CV/%	4.98	8.89	15.09	5.97	13.82	14.01	13.80			
	Germination rate%	Average	94.89 a	82.00 b	75.56 c	92.00 a	77.56 c	82.89 b	84.15	68.45 **	37.69 **	7.88 **
		CV/%	3.99	8.91	13.83	5.23	13.02	14.21	13.21			
	Germination Index	Average	45.92 a	37.20 b	33.89 c	44.17 a	35.78 b	37.07 b	39.01	78.23 **	41.33 **	10.70 **
		CV/%	5.05	9.75	21.33	8.04	16.14	21.11	18.00			
	Vigor index	Average	609.39 a	440.41 b	421.96 b	585.22 a	417.55 c	469.00 b	490.59	133.97 **	92.62 **	13.02 **
		CV/%	7.91	17.44	25.49	11.10	23.40	24.46	24.03			

Note: The same lowercase letters after averages show that the difference between different varieties or environments has not reached the significance level of 0.05; * and ** indicate a significant correlation at the level of 0.05 and 0.01, respectively (the same below).

## Data Availability

The original contributions presented in the study are included in the article/Supplementary Materials; further inquiries can be directed to the corresponding author.

## Supplementary Materials

The following supporting information can be downloaded at: https://www.mdpi.com/article/10.3390/foods14060982/s1, Figure S1: Statistics of common and unique OTUs of endophytes in wheat grains (Note: Bacterial OTUs across three wheat varieties (A) and three cultivation environments (B); fungal OTUs across three wheat varieties (C) and three cultivation environments (D)); Figure S2: Statistics of common and unique OTUs of Daqu microorganisms (Note: Bacterial OTUs across three fermentation stages (A), three cultivation environments (B), and three wheat varieties (C); fungal OTUs across three fermentation stages (D), three cultivation environments (E), and three wheat varieties (F)).
