# Supplementary figures and images for: Effect of Wheat Varieties and Cultivation Environments on Grain Endophytes, Microbial Communities, and Quality of Medium-High Temperature Daqu in Chinese Baijiu
Source: Foods. 2025 Mar 13;14(6):982. doi: 10.3390/foods14060982 (PMC11940993; doi:10.3390/foods14060982)

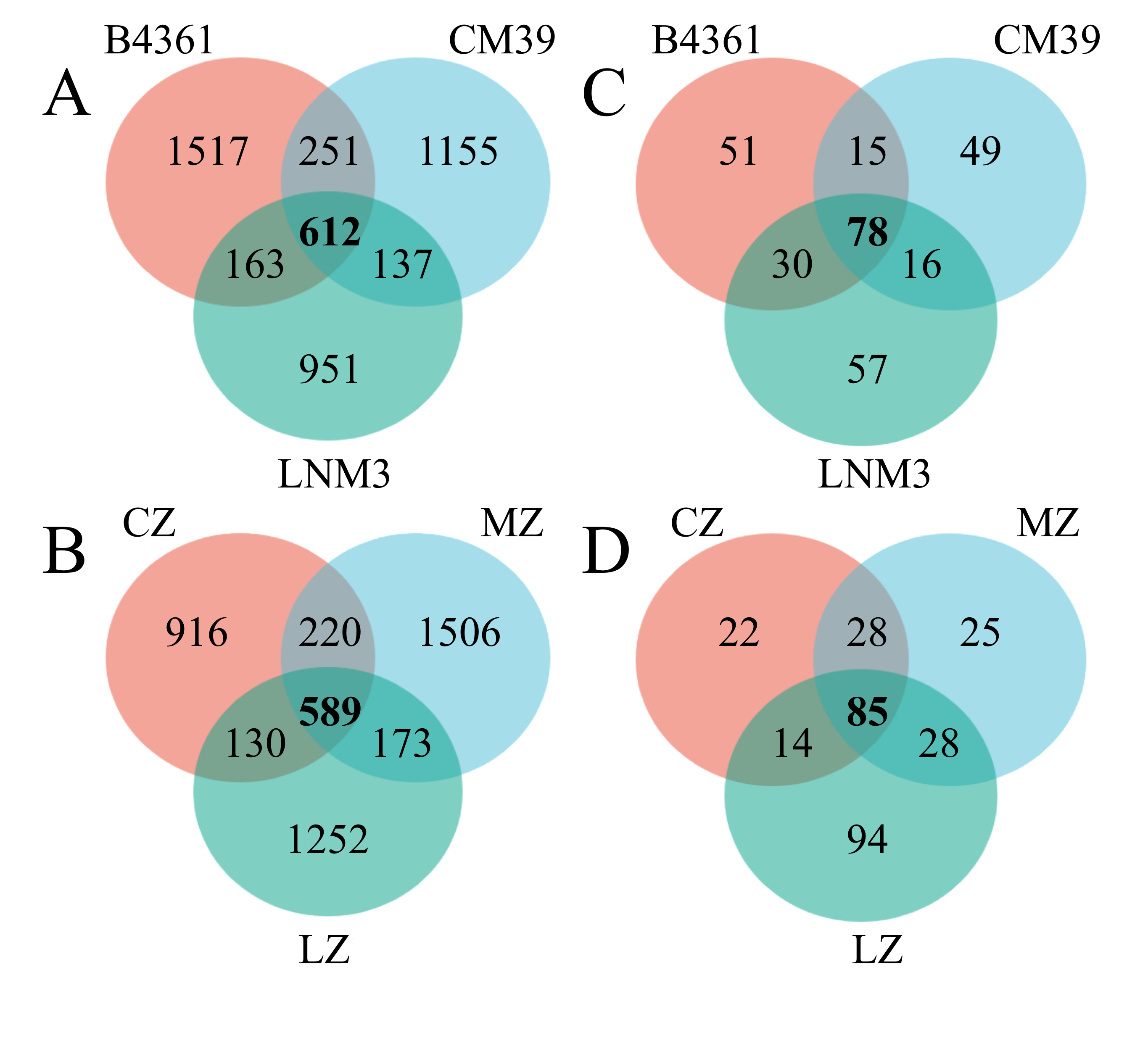

Supplement: Supplementary file 1 [file foods-14-00982-s001.zip › Fig. S1.tif]

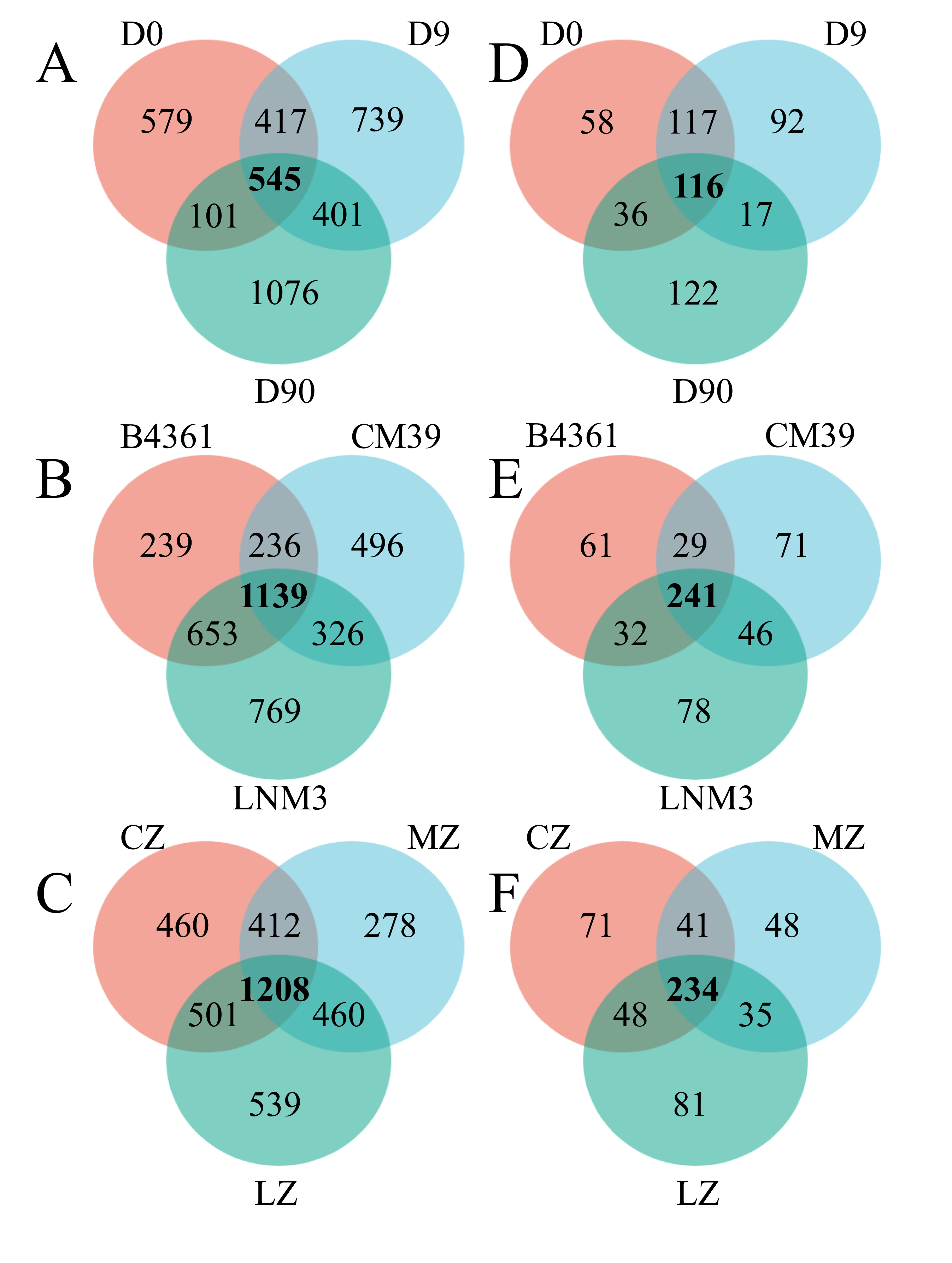

Supplement: Supplementary file 1 [file foods-14-00982-s001.zip › Fig. S2.tif]
